# Blockade of PD-1 Signaling Enhances Th2 Cell Responses and Aggravates Liver Immunopathology in Mice with Schistosomiasis japonica

**DOI:** 10.1371/journal.pntd.0005094

**Published:** 2016-10-28

**Authors:** Sha Zhou, Xin Jin, Yalin Li, Wei Li, Xiaojun Chen, Lei Xu, Jifeng Zhu, Zhipeng Xu, Yang Zhang, Feng Liu, Chuan Su

**Affiliations:** Department of Pathogen Biology and Immunology, Jiangsu Key Laboratory of Pathogen Biology, Nanjing Medical University, Nanjing, Jiangsu, China; University of Manchester, UNITED KINGDOM

## Abstract

**Background:**

More than 220 million people worldwide are chronically infected with schistosomes, causing severe disease or even death. The major pathological damage occurring in schistosomiasis is attributable to the granulomatous inflammatory response and liver fibrosis induced by schistosome eggs. The inflammatory response is tightly controlled and parallels immunosuppressive regulation, constantly maintaining immune homeostasis and limiting excessive immunopathologic damage in important host organs. It is well known that the activation of programmed death 1 (PD-1) signaling causes a significant suppression of T cell function. However, the roles of PD-1 signaling in modulating CD4^+^ T cell responses and immunopathology during schistosome infection, have yet to be defined.

**Methodology/Principal Findings:**

Here, we show that PD-1 is upregulated in CD4^+^ T cells in *Schistosoma japonicum* (*S*. *japonicum*)-infected patients. We also show the upregulation of PD-1 expression in CD4^+^ T cells in the spleens, mesenteric lymph nodes, and livers of mice with *S*. *japonicum* infection. Finally, we found that the blockade of PD-1 signaling enhanced CD4^+^ T helper 2 (Th2) cell responses and led to more severe liver immunopathology in mice with *S*. *japonicum* infection, without a reduction of egg production or deposition in the host liver.

**Conclusions/Significance:**

Overall, our study suggests that PD-1 signaling is specifically induced to control Th2-associated inflammatory responses during schistosome infection and is beneficial to the development of PD-1-based control of liver immunopathology.

## Author Summary

Schistosomiasis is a parasitic disease that affects approximately 220 million people and causes serious morbidity and economic problems mainly in (sub)tropical regions. After *Schistosoma japonicum* or *Schistosoma mansoni* infection, parasite eggs are trapped in host liver and induce liver inflammation and fibrosis, leading to irreversible impairment of the liver, and even death of the host. Meanwhile, schistosomes also induce strong regulatory mechanisms to suppress inflammation and prevent excessive immunopathology. Considering it is well known that PD-1 plays a critical role in suppressing T cell function, understanding the role of PD-1 in modulating immune responses during schistosome infection is necessary for the development of PD-1-based control of liver damage in schistosomiasis. Here, increased PD-1 expression in CD4^+^ T cells from both humans and mice with schistosome infection was shown. We further showed that PD-1 blockade preferentially augmented Th2 cell responses and ultimately resulted in more severe liver immunopathology in mice with Schistosomiasis japonica, suggesting that PD-1 signaling is beneficial to further explore therapeutic possibilities for preventing the excessive liver immunopathology.

## Introduction

Schistosomiasis is an infectious disease that affects at least 220 million people worldwide and causes serious morbidity and economic problems in developing countries [1,2]. During infection with *Schistosoma japonicum* (*S*. *japonicum*) or *S*. *mansoni*, granulomas form around eggs that are trapped in the host liver. This long-term immune-mediated granulomatous response results in severe fibrosis in the liver and eventually causes extensive tissue scarring, leading to irreversible impairment of affected organs, particularly the liver, and even death of the host [3–5].

The CD4^+^ T cell subsets play a critical role to develop hepatic granulomas and to maintain a balanced granulomatous response to prevent the growth of hepatic fibrosis during schistosomiasis [6,7]. Meanwhile, schistosomiasis also induces strong regulatory mechanisms, including T cell hyporesponsiveness, to prevent excessive immunopathology [8].

The inhibitory receptor programmed cell death 1 (PD-1) is expressed in activated T cells and functions as a pivotal immune checkpoint protein that plays a critical role in the regulation of T cell function as well as its dysfunction in certain contexts [9–11]. Increasingly, studies in a number of murine and human infectious disease models and cancers have found an immunoregulatory function for PD-1 in T cells [12–17]. Recently, numerous studies have shown that exploiting the PD-1 pathway may be of interest for the treatment of chronic viral infections, cancers and autoimmune diseases [12–14]. PD-1 ligand 1 and 2 (PD-L1/L2) have been shown to be significantly upregulated in macrophages and dendritic cells during schistosome infection, suggesting their involvement in T cell anergy [18,19]. However, very little is known about the regulation of PD-1 in CD4^+^ T cells or the impact of its signaling on the development of CD4^+^ T cell responses and egg-induced immunopathology during schistosome infections.

In this study, we show that PD-1 expression is significantly up-regulated in CD4^+^ T cells from both humans and mice with schistosome infection. We further found that the inhibition of PD-1 signaling specifically enhanced T helper 2 (Th2) cell responses and ultimately led to more severe liver immunopathology in mice with Schistosomiasis japonica.

## Methods

### Ethics statement

All the animal experiments were conducted in strict accordance with the Regulations for the Administration of Affairs Concerning Experimental Animals (1988.11.1), and all efforts were made to minimize suffering. All the animals were used with approval by the Institutional Animal Care and Use Committee (IACUC) of Nanjing Medical University for the use of laboratory animals (Permit Number: NJMU 14–0711).

Ethical approval for the human blood samples used in this study was obtained from the Institutional Review Board of Nanjing Medical University, Nanjing, China (Permit Number: 2014NMUIEC001). Written informed consent was obtained from each participant. Individuals with positive stool examination results were treated with a single oral dose of praziquantel (40 mg/kg). All personal identifiers of the study notes and tapes were kept confidential and destroyed after the study was completed.

### Patients and healthy controls

A total of 43 subjects were enrolled in the study. These subjects were from a village in Chizhou City, Anhui province. The subjects included 13 healthy adult controls and 26 patients with schistosomiasis japonica, diagnosed by the detection of parasite eggs using the Kato-Katz method with duplicate examination of three consecutive stool specimens obtained from each individual [20]. The healthy controls displayed no history, laboratory or clinical signs of schistosome infection. Participants who were positive for other intestinal helminth infections in the egg detection were excluded from this study. Furthermore, all of the participants were interviewed in person at enrollment. Participants who had been infected by hepatitis virus or had a history of influenza virus infection within 4 weeks were excluded from this study.

### Mice and infection

Specific pathogen-free (SPF) 8-wk-old female C57BL/6 mice were purchased from the Model Animal Research Center of Nanjing University (Nanjing, China). All the mice were housed and handled in accordance with the guidelines of Chinese animal protection laws with permission from the Institutional Review Board of Nanjing Medical University.

Each C57BL/6 mouse was percutaneously infected with 12 cercariae of the Chinese mainland strain of *S*. *japonicum* from infected snails (*Oncomelania hupensis*) acquired from the Jiangsu Institute of Parasitic Diseases (Wuxi, China).

### Preparation of SEA/SWA (soluble egg antigens/soluble worm antigens)

*S*. *japonicum* SEA and SWA were prepared as previously described [21,22]. The antigens were filter-sterilized and endotoxin was removed using Polymyxin B-Agarose (Sigma-Aldrich, St. Louis, MO). The endotoxin activity (<0.01 EU/μg) was determined using the LAL assay kit (BioWhittaker, Walkersville, MD). Protein concentrations were determined using the Lowry method (DC Protein Assay Kit, Bio-Rad, Hercules, CA).

### Immunofluorescence staining and flow cytometry (FCM)

Human peripheral blood mononuclear cells (PBMCs) were separated from whole blood by Ficoll-Paque PLUS (GE healthcare, Uppsala, Sweden) density gradient centrifugation. Cells were recovered from the gradient interface, washed twice and stained for 30 min at 4°C with the following antibodies: CD3-FITC (clone HIT3a), CD4-PerCP-Cy5.5 (clone RPA-T4), PD-1-PE-Cy7 (clone EH12.1), all from BD Biosciences (San Jose, CA). For measurement of Foxp3 expression, cells were further permeabilized at room temperature, incubated for 15 min at 4°C in permeabilization buffer containing anti-FcγR (eBioscience, San Diego, CA) to avoid nonspecific binding, and then stained for 30 min at 4°C with Foxp3-PE (clone 259D/C7, BD Biosciences).

Spleens and mesenteric lymph nodes (LNs) were extracted from mice and pressed through nylon nets to prepare single-cell suspensions. Following red blood cell lysis, the remaining cells were washed and counted. Single cell suspensions of hepatic lymphocytes were prepared as previously described [23–25]. To analyze PD-1 expression in CD4^+^ T cells, the cells were incubated with CD3-APC (clone 145-2C11), CD4-FITC (clone RM4-5) and PD-1-PE/PE-Cy7 (clone J43, all from eBioscience). To determine intracellular cytokine expression, T cells from each mouse were stimulated with 25 ng/ml of phorbol myristate acetate (PMA; Sigma-Aldrich, St. Louis, MO) and 1 μg/ml of ionomycin (Sigma-Aldrich) in complete RPMI 1640 medium (Gibco, Grand Island, NY) in the presence of 1 μl/ml of Golgistop (BD PharMingen, San Diego, CA) for 6 h at 37°C in 5% CO_2_. After 6 h, the cells were collected and surface stained with CD3-APC (clone 145-2C11) and CD4-FITC (clone RM4-5), and washed, fixed and permeabilized with Cytofix/Cytoperm buffer (BD PharMingen). Next, the cells were intracellularly stained with PE-conjugated antibodies against IFN-γ (clone XMG1.2), IL-4 (clone 11B11), IL-17A (clone eBio17B7), or rat IgG1 isotype antibody (all from eBioscience) as a control. To analyze regulatory T cells, the Mouse Regulatory T Cell Staining Kit (eBioscience) was used, and the cells were surface stained with CD3-PerCP-Cy5.5 (clone 145-2C11), CD4-FITC (clone RM4-5), and CD25-APC (clone PC61.5). The cells were then permeabilized with cold Fix/Perm Buffer, and the Fc receptors were blocked with anti-mouse CD16/32 (Fc Block) for 15 min. A PE-labeled anti-mouse Foxp3 (clone FJK16s) or rat IgG2a isotype control antibody was then added.

Following immunofluorescence staining, the cells were examined using a FACS Verse instrument (BD Bioscience) and analyzed using FlowJo (Tree Star, version 10.0.7). The cells were gated on CD3^+^CD4^+^ T cells.

### Cell culture

Single cell suspensions of splenocytes were prepared from the spleens of SPF C57BL/6 mice. The splenocytes were then stimulated *in vitro* with SWA (20 μg/ml), SEA (20 μg/ml), or PBS as a control. The cells were cultured in triplicate with complete RPMI 1640 medium (Gibco, Grand Island, NY) in 96-well round-bottom culture plates (1.5×10^6^ cells/ml) for 3 d and then collected for FCM analysis.

### *In vivo* blocking Ab administration

To block PD-1 *in vivo*, 100 μg of rat anti-mouse PD-1 mAb (clone 29F.1A12; Biolegend, San Diego, CA), rat IgG2a isotype control (Biolegend) or PBS was injected intraperitoneally (i.p.) every three days, starting 24 d post-infection until 3 d before the mice were sacrificed [26]. At 42 d post-infection, all the mice were sacrificed. Serum samples were collected for ELISA detection of IL-4 levels, and splenocytes were prepared for FCM and qPCR analysis. In addition, livers were isolated for a pathological examination and an assessment of their egg burden.

### Quantitative real-time PCR (qPCR)

Total RNA from mouse splenocytes was prepared using TRIzol reagent (Invitrogen, Carlsbad, CA). Any potentially contaminating DNA was removed using on-column DNAse treatment (Qiagen, Hilden, Germany). An equivalent amount of total RNA from each sample was reverse transcribed with random hexamers using the SuperScript III First-Strand cDNA Synthesis System (Invitrogen). The synthesized cDNA samples were then used as templates for qPCR and performed on a 7300 Real-Time PCR System (Applied Biosystems, Foster City, CA) with FastStart SYBR Green Master Mix (Roche Applied Science, Meylan, France). Gene expression levels were normalized and shown as the fold increase over control. The following primers were used:

IFN-γ, forward, 5’- TGCTGATGGGAGGAGATGTCT-3’, and reverse, 5’- TGCTGTCTGGCCTGCTGTTA-3’;

IL-12 p35, forward, 5’-GATGCAGTCTCTCTGAATCATAATGG -3’, and reverse, 5’-GGCACAAAAACAATAGCTTATCAGT -3’;

IL-4, forward, 5’-ACAGGAGAAGGGACGCCAT-3’, and reverse, 5’-GAAGCCCTACAGACGAGCTCA-3’;

IL-13, forward, 5’- CCTGGCTCTTGCTTGCCTT-3’, and reverse, 5’- GGTCTTGTGTGATGTTGCTCA-3’;

IL-17A, forward, 5’-TCAGCGTGTCCAAACACTGAG-3’, and reverse, 5’-CGCCAAGGGAGTTAAAGACTT-3’;

IL-23, forward, 5’- AATAATGTGCCCCGTATCCAGT-3’, and reverse, 5’- GCTCCCCTTTGAAGATGTCAG-3’;

IL-10, forward, 5’-ACTTTAAGGGTTACTTGGGTTGC-3’, and reverse, 5’-ATTTTCACAGGGGAGAAATCG-3’;

TGF-β1, forward, 5’-ATGCTAAAGAGGTCACCCGC-3’, and reverse, 5’-CCAAGGTAACGCCAGGAATT-3’;

GAPDH, forward, 5’-TGGTGAAGGTCGGTGTGAAC-3’, and reverse, 5’-CCATGTAGTTGAGGTCAATGAAGG-3’.

### Serum IL-4 analysis

The levels of IL-4 in serum were determined using a commercial ELISA kit (Dakewe, Shenzhen, China) according to the manufacturer’s instructions. The cytokine concentration in each sample was extrapolated from a standard curve.

### Liver pathology

Liver tissue from infected mice was fixed in 4% buffered formalin, embedded in paraffin and sectioned (5–7 μm). The liver sections were stained with hematoxylin and eosin (H&E) to determine the size of granulomas. For each mouse, the sizes of 30 granulomas around individual eggs were quantified with the AxioVision Rel 4.7 Imaging System (Zeiss, Oberkochen, Germany). The data are expressed in area units. All the images were captured at 100× magnification using an Axiovert 200M microscope and analyzed with Axiovision software (Zeiss).

The liver sections were stained with 0.1% Sirius red (Sigma-Aldrich) for semi-quantitative analysis of hepatic fibrosis [27]. Six to eight fields from each slide were randomly obtained using an optical microscope (Zeiss) coupled with a digital camera. The red-stained area per total area and the intensity of fibrosis were measured using Image-Pro Plus software (version 6.0 for Windows; Media Cybernetics, Rockville, MD). A total fibrosis density score was determined by dividing the image intensity by the image area. Intensity exclusion parameters were identical for each of the images captured.

### Assessment of egg burden

Two grams of liver tissue from each infected mouse was digested with 5% KOH at 37°C overnight, and then the number of eggs per gram of liver was determined by microscopic examination.

### Statistical analysis

Significant differences were assessed using the SPSS program (version 11.0 for Windows; SPSS, Inc., Chicago, IL). The comparisons between two groups were analyzed by Student’s *t*-test. Comparisons between more than two groups were analyzed with one-way analysis of variance (ANOVA) using an LSD post hoc test. *P* values comparing human data were calculated using Chi-Square Test for categorical variables, and Mann-Whitney *U* test or Student’s *t*-test for continuous variables. *P* < 0.05 was considered to be statistically significant.

## Results

### PD-1 expression is elevated in CD4^+^ T cells from *S*. *japonicum*-infected patients

**Fig 1:**
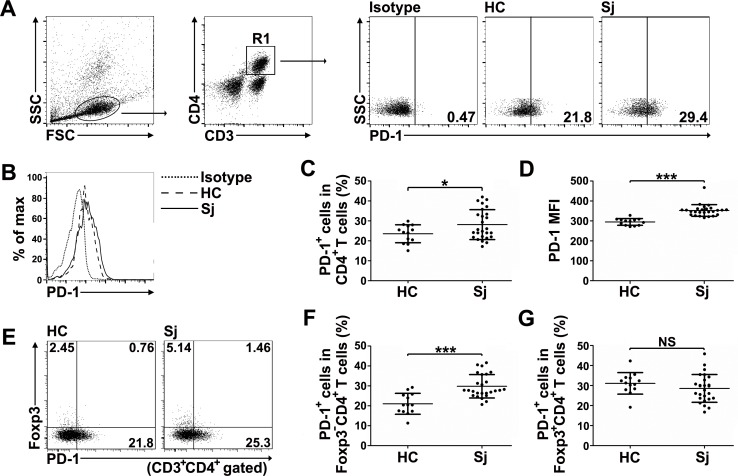
PD-1 expression is elevated in CD4^+^ T cells from *S*. *japonicum*-infected patients. (A) The polychromatic flow cytometry gating scheme for the identification of CD3^+^CD4^+^PD-1^+^ T cells in PBMCs is shown. The gating strategy shows total CD3^+^CD4^+^ T cells (R1). Representative dot plots show PD-1 expression in CD4^+^ T cells. (B) Overlay of representative histograms showing PD-1 expression in CD4^+^ T cells. (C) Pooled data from two independent experiments showing the percentage of CD4^+^ T cells expressing PD-1 in total CD4^+^ T cells from healthy donors (n = 13) or schistosomiasis patients (n = 26). (D) Pooled data from two independent experiments showing the MFI of PD-1 expression in CD4^+^ T cells from healthy donors (n = 13) or schistosomiasis patients (n = 26). (E) Representative dot plots show PD-1 expression within Foxp3^-^CD4^+^ T cells or Foxp3^+^CD4^+^ T cells. (F and G) Pooled data from two independent experiments showing the average percentage of PD-1-expressing Foxp3^-^CD4^+^ T cells or Foxp3^+^CD4^+^ T cells from healthy donors (n = 13) or schistosomiasis patients (n = 26). HC, healthy control; *Sj*, *Schistosomiasis japonica*. Each dot represents an individual, and horizontal lines depict the mean values (C, D). Error bars indicate SD. *P < 0.05, ***P < 0.001.

It is currently unknown if PD-1 is induced in CD4^+^ T cells in schistosomiasis patients. Therefore, we assessed the expression of PD-1 in CD4^+^ T cells from the peripheral blood of *S*. *japonicum*-infected patients. Total of 26 patients and 13 healthy controls were recruited and there was no statistically significant difference in the distribution of age or gender between groups (Table 1). The gating scheme for the identification of the human CD4^+^PD-1^+^ T cell population is shown in Fig 1A. Overall, a greater number of CD4^+^ T cells in *S*. *japonicum*-infected patients expressed PD-1 than in healthy controls (Fig 1A–1C). As shown in Fig 1D, similar patterns were observed when PD-1 expression was analyzed by mean fluorescence intensity (MFI). In addition, results showed that PD-1 expression increased in Foxp3^-^CD4^+^ T cells rather than in Foxp3^+^CD4^+^ T cells (Fig 1E–1G). Collectively, our results demonstrate that PD-1 expression is upregulated in CD4^+^ T cells from *S*. *japonicum*-infected patients.

**Table 1:** The demographic and clinical characteristics of subjects.

Parameters	HC	Sj	P-value
Number	13	26	
Age (years)			
Mean ± SD	63.2 ± 9.0	65.3 ± 7.4	> 0.05
	(48~72)	(47~74)	(Mann-Whitney *U* test)
Sex N (%)			
Male	7 (53.9%)	16 (61.5%)	> 0.05
Female	6 (46.1%)	10 (38.5%)	(Pearson Chi-Square Test)

HC, healthy control; Sj, Schistosomiasis japonica.

### PD-1 expression is elevated in CD4^+^ T cells from *S*. *japonicum*-infected mice

**Fig 2:**
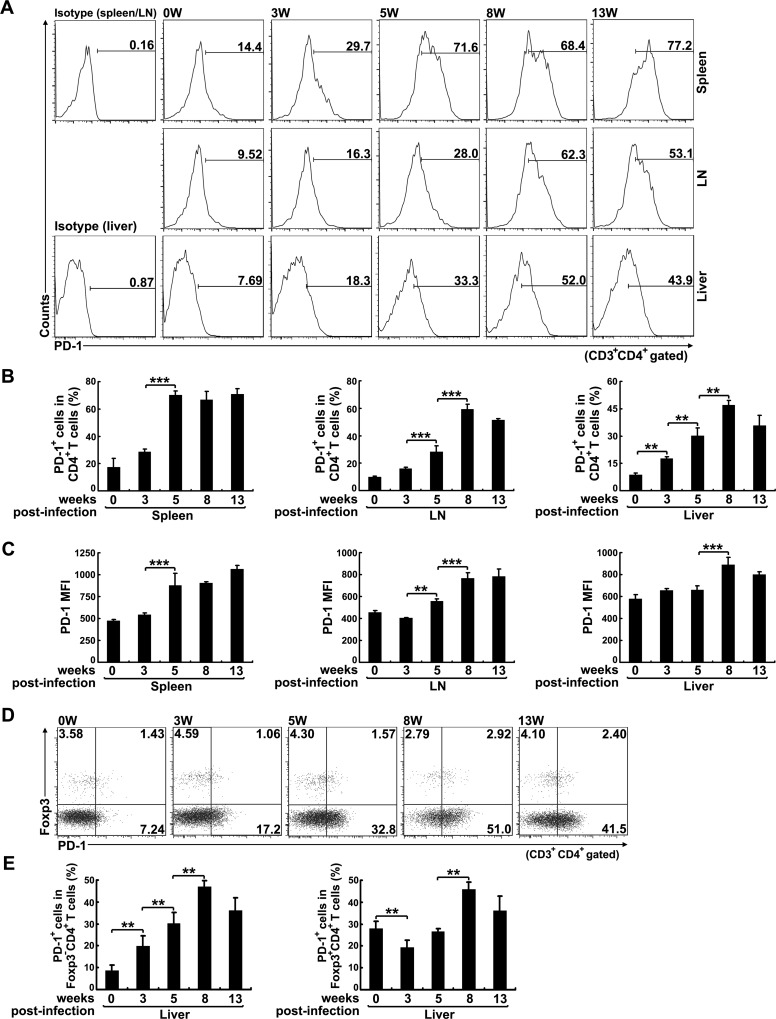
PD-1 expression is elevated in CD4^+^ T cells from *S*. *japonicum*-infected mice. (A) CD3^+^CD4^+^PD-1^+^ T cells in mouse splenocytes, mesenteric or hepatic lymphocytes were analyzed by FCM. Representative histograms show PD-1 expression in CD4^+^ T cells from randomly selected mice that were sacrificed at 0 (before infection), 3, 5, 8 and 13 weeks post-infection. (B) The bar graphs show the average percentages of CD4^+^ T cells expressing PD-1 in total mouse CD4^+^ T cells. (C) The bar graphs show the average MFI of PD-1 expression in mouse CD4^+^ T cells. (D) PD-1 expression on Foxp3^-^CD4^+^ T cells or Foxp3^+^CD4^+^ T cells in hepatic lymphocytes from *S*. *japonicum*-infected mice was analyzed by FCM at indicated time points post-infection. Representative dot plots illustrating PD-1 expression within Foxp3^-^CD4^+^ T cells or Foxp3^+^CD4^+^ T cells. (E) The bar graphs show the average percentages of PD-1-expressing Foxp3^-^CD4^+^ T cells or Foxp3^+^CD4^+^ T cells. Data are expressed as the means ± SD of 5 mice. Similar results were obtained in two or three independent experiments. **P < 0.01, ***P < 0.001.

Next, we observed the kinetics of PD-1-expressing CD4^+^ T cells, as well as the relative PD-1 MFI, at different time points following *S*. *japonicum* infection in mice. Both the frequency and MFI of PD-1 expression in splenic and mesenteric CD4^+^ T cells showed a continuous increase after infection (Fig 2A–2C). Specifically, PD-1 expression in splenic CD4^+^ T cells barely increased during the first three weeks post-infection and then rapidly increased (average fold-increase of 4.1 in frequency, 5.6 in total number, and 2 in MFI) and reached a plateau at five weeks post-infection, remaining at a high level thereafter (Fig 2B and 2C and S1 Fig). The mesenteric CD4^+^ T cells showed a similar, but slightly slower, increase in PD-1 expression (average fold-increase of 2.9 in frequency, 1.8 in total number, and 1.2 in MFI) during the first five weeks post-infection, reaching a plateau at eight weeks post-infection (average fold-increase of about 6 in frequency, 5.6 in total number, and 1.7 in MFI) (Fig 2B and 2C and S1 Fig). Meanwhile, the CD4^+^ T cells in liver showed a continuous increase in PD-1 expression (frequency and MFI) since three weeks post-infection and reached a plateau at eight weeks post-infection (Fig 2A–2C). These results demonstrate that the expression of PD-1 increases in CD4^+^ T cells after *S*. *japonicum* infection. In addition, Foxp3^-^CD4^+^ T cells showed a continuous increase in PD-1 expression till eight weeks post-infection. However, PD-1 expression in Foxp3^+^CD4^+^ T cells was significantly decreased at three weeks post-infection and increased since eight weeks post-infection (Fig 2D and 2E). We also detected PD-1 expression on non-CD4^+^ T cells in *S*. *japonicum*-infected mice and found that the frequency of PD-1 expression on splenic CD8^+^ T cells or non-T cells (CD3^-^ cells) (S2 Fig) was much lower than that on splenic CD4^+^ T cells (Fig 2A and 2B).

To determine whether CD4^+^ T cells are liable to be anergic, we analyzed Fas and PD-L1 expression by FCM. Compared with normal uninfected control mice, significantly higher levels of Fas and PD-L1 were detected on splenic and mesenteric CD4^+^ T cells of *S*. *japonicum*-infected mice eight weeks post-infection, suggesting that CD4^+^ T cells tend to be anergic in *S*. *japonicum* infection (S3 Fig).

### PD-1 blockade augments Th2 cell responses in *S*. *japonicum*-infected mice

**Fig 3:**
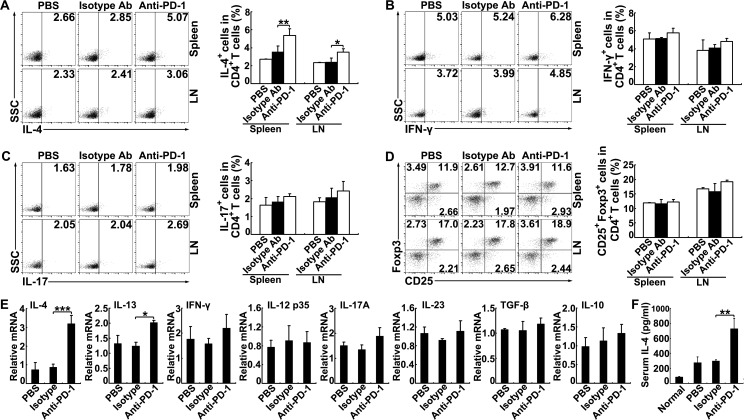
PD-1 blockade augments Th2 cell responses in *S*. *japonicum*-infected mice. (A-D) Representative staining and mean percentages for IL-4-, IFN-γ-, IL-17A-producing CD4^+^ T cells (A-C) and Treg cells (D) from the spleens and LNs of *S*. *japonicum*-infected mice treated with anti-PD-1 mAb, control rat IgG2a or PBS. Data are expressed as the means ± SD of 5 mice. Similar results were obtained in two or three independent experiments. *P < 0.05, **P < 0.01. (E) Relative mRNA expression levels of IL-4, IL-13, IFN-γ, IL-12 p35, IL-17A, IL-23, TGF-β and IL-10 in splenocytes from *S*. *japonicum*-infected mice treated with anti-PD-1 mAb or control rat IgG2a. (F) Total serum IL-4 levels in mice were measured by ELISA. Data are expressed as the means ± SD of 5 mice. Similar results were obtained in two or three independent experiments. *P < 0.05, **P < 0.01, ***P < 0.001.

The CD4^+^ T cell subsets are involved in the regulation of schistosomiasis progression [3]. FCM analyses revealed significantly increased frequencies and numbers of IL-4-producing splenic and mesenteric CD4^+^ T cells in *S*. *japonicum*-infected mice treated with a blocking anti-PD-1 mAb (Fig 3A and S4A Fig). However, the frequencies or numbers of IFN-γ^+^ population (Fig 3B and S4B Fig), IL-17A^+^ population (Fig 3C and S4C Fig), and Treg cells (Fig 3D and S4D Fig) in CD4^+^ T cells did not show any significant increase after the PD-1 blockade. Similar results were also obtained in liver (S5 Fig). Additionally, there were also no significant differences in the proportions of activated (CD62L^low^CD44^hi^) or resting Treg cells (CD62L^hi^CD44^low^) from either spleens or LNs among groups (S6 Fig).

To investigate whether PD-1 restricts Th2 effector function or Th2 differentiation, we detected GATA-3 level in CD4^+^ T cells. As shown in S7A and S7B Fig, PD-1 blockade did not affect GATA-3 expression in splenic or mesenteric CD4^+^ T cells from *S*. *japonicum*-infected mice, suggesting PD-1 does not affect Th2 differentiation but regulates Th2 effector function. On the other hand, no significant change of PD-1 expression was detected in GATA-3^+^CD4^+^ T cells after PD-1 blockade (S7A and S7C Fig).

Consistently, PD-1 blockade in infected mice resulted in significantly increased mRNA expression of the Th2 (IL-4 and IL-13) but not Th1 (IFN-γ and IL-12), Th17 (IL-17 and IL-23) or Treg (TGF-β and IL-10) -associated cytokines in splenocytes from *S*. *japonicum*-infected mice (Fig 3E). We next examined the systemic levels of IL-4 in the serum of infected mice with or without PD-1 blockade. We found that the levels of serum IL-4 were significantly greater in mice that received PD-1 blockade than in control mice (Fig 3F). Consistently, PD-1 blockade in infected mice significantly increased the frequency of M2 macrophages in liver (S8 Fig). Thus, PD-1 blockade promoted Th2 cell responses, suggesting that PD-1 may restrict Th2 cell responses during *S*. *japonicum* infection.

### PD-1 blockade enhances hepatic immunopathology in *S*. *japonicum*-infected mice

**Fig 4:**
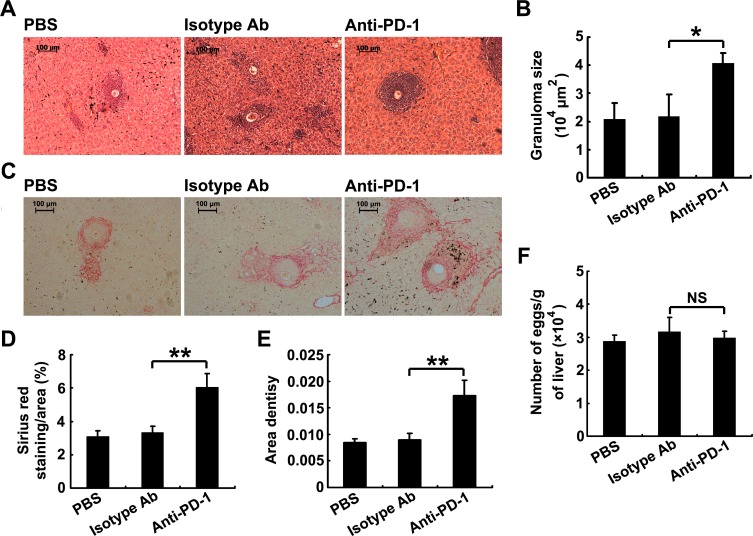
PD-1 blockade enhances hepatic immunopathology in *S. japonicum*-infected mice. (A) Infected mice were treated with PBS, anti-PD-1 mAb or control rat IgG2a. Liver sections were H&E stained to reveal granulomas (original magnification, 100×). Images are representative of three independent experiments. (B) The mean area of granulomas around individual eggs was measured. (C) Liver sections were stained with Sirius red to reveal granulomas (original magnification, 100×). (D and E) Quantification of Sirius red staining was performed using Image-Pro Plus software and is represented as the percentage of the stained-area per total area (D) and area density (E). (F) The number of eggs extracted from the livers of mice was determined by microscopic examination. Protection was measured by assessing the egg burden. Data are expressed as the means ± SD of 5 mice. Similar results were obtained in three independent experiments. *P < 0.05, **P < 0.01, NS, not significant.

Previous studies have shown that stronger Th2 cell responses during *S*. *japonicum* infection result in more severe hepatic immunopathology [6,7]. The results in Fig 4A and 4B show that the average liver granuloma size in infected mice receiving anti-PD-1 mAb treatment was significantly increased compared to the granulomas in control mice. In addition, PD-1 blockade enhanced the severity of liver fibrosis in infected mice (Fig 4C–4E). In addition, compared to the control group, no reduction of egg burden was observed in the livers of infected mice receiving anti-PD-1 mAb treatment (Fig 4F). Thus, PD-1 blockade results in enhanced immunopathology in *S*. *japonicum*-infected mice.

## Discussion

Multiple immunoregulatory mechanisms are triggered by schistosomes to protect the host from severe immunopathology [3,7,8]. PD-1 signaling plays a critical role in the regulation of T cell function, as well as its dysfunction in certain contexts [9–11]. However, the role of PD-1 in schistosome infections remains elusive. Here, we uncovered that the PD-1 pathway specifically enhances Th2 cell responses and is critical to control liver immunopathology in mice with Schistosomiasis japonica.

Previous studies have demonstrated that along with T cell suppression during schistosomal infection, the expression of PD-L1 and PD-L2 are selectively up-regulated in macrophages and dendritic cells respectively [18,19], suggesting critical roles for both PD-L1 and PD-L2 in regulating T cell responses during schistosomal infection. However, the role of PD-L1/L2 in the regulation of CD4^+^ T cell responses and egg-induced immunopathology during schistosomal infections have not yet been investigated. To our knowledge, the present study is the first to report a significantly higher expression of PD-1 in CD4^+^ T cells from chronic schistosomiasis patients. In consistence with previous report [19], we also observed a gradual increase in PD-1 expression in CD4^+^ T cells *in vivo*. Additionally, both splenic and mesenteric CD4^+^ T cells had a high expression level of PD-1, even 8 weeks post-infection. This may, in part, account for the hyposensitive phenotype of CD4^+^ T cells observed in the later stages of chronic schistosomiasis [8]. Together, these observations suggest that increased PD-1 expression may be instrumental in the modulation of CD4^+^ T cell immune responses during chronic infection.

To support this hypothesis, blocking antibodies against PD-1 were examined in *S*. *japonicum*-infected mice. The blockade of the PD-1 pathway in *S*. *japonicum*-infected mice selectively enhanced Th2 cell responses by increasing Th2 cells and the levels of Th2-type cytokines (IL-4 and IL-13), suggesting that the PD-1 pathway controls the Th2 cell responses during schistosome infection. Although PD-1/PD-L1 signaling has been reported to be involved in the development or proliferation of regulatory T cells in PD-L1^-/-^ mice models or patients with chronic virus infection [28,29], similar numbers of Tregs were observed in the spleens and lymph nodes of *S*. *japonicum*-infected mice receiving PD-1 blocking antibodies. Thus, this finding is inconsistent with prior studies [28,29] and suggests that the PD-1 pathway may be redundant for the peripheral induction of Treg cells during schistosome infection. Overall, our present study is the first to suggest that PD-1 blockade selectively augments Th2 cell responses in the spleens, mesenteric lymph nodes, or livers of mice with schistosomal infection, though the mechanism by which this occurs remains unclear. Further studies will be important to better understand how the PD-1 pathway regulates Th2 cell responses during chronic helminthic infections.

It has been previously reported that the development of pathology during schistosome infections is typically driven by Th2 immune responses [6,7], suggesting that PD-1 may limit this immunopathology by inhibiting Th2 cell responses. Indeed, we blocked PD-1 signaling and observed that mice infected with *S*. *japonicum* suffered more severe liver pathology, demonstrating the importance of the PD-1 pathway to reduce liver immunopathology during chronic schistosome infections. The PD-1 pathway has also been shown to be associated with long-term exposure to schistosome eggs and elevated Th2 responsiveness to SEA [30]. However, considering PD-1 blocking antibodies may target all populations of PD-1-expressing cells, it is definitely possible that some other PD-1-expressing cells, except for CD4^+^ T cells, may also be involved in the regulation of liver immunopathology after schistosome infection.

However, in contrast to many studies that support a dominant role for PD-1 blockade in protecting against infection [15–17,28], here, we found that PD-1 blockade failed to elicit protection against schistosomes in mice, with no reduction of the schistosome egg burden. One possible reason is that immune protection against schistosomes is associated with the induction of Th1-biased immune responses [30–32]. However, in our study, PD-1 blockade had no effect on Th1 immune responses. Overall, our results suggest that the PD-1-mediated reduction of hepatic immunopathology during schistosome infection is due to its immune regulation, not a reduction in egg burden.

Taken together, our study is the first to demonstrate that egg antigens are likely responsible for the upregulation of PD-1 in CD4^+^ T cells in mice with *S*. *japonicum* infection. This results in a specific suppression of the Th2 cell response and leads to reduced liver immunopathology in mice during schistosome infection. It will be of interest to further explore therapeutic possibilities that target this inhibitory PD-1/Th2 axis for preventing the excessive immunopathology caused by an overactive immune response to schistosome infection.

## Supporting Information

S1 Fig The total number of PD-1^+^CD4^+^ T cells is increased in the spleens or LNs of *S*. *japonicum*-infected mice. The bar graphs show the absolute number of PD-1^+^CD4^+^ T cells in splenic or mesenteric cells from mice at indicated time points after *S*. *japonicum* infection. The absolute numbers of PD-1^+^CD4^+^ T cells were calculated as following: total cell number of the splenic or mesenteric cells × (frequency of CD4^+^ T cells in total cells) × (frequency of PD-1^+^ cells in total CD4^+^ T cells). The data are expressed as the means ± SD of 15 mice from three independent experiments. *P < 0.05, **P < 0.01, ***P < 0.001.
(TIF)

S2 Fig PD-1 expression on CD8^+^ T cells or CD3^-^ non-T cells in *S*. *japonicum*-infected mice. (A) PD-1 expression on CD8^+^ T cells or CD3^-^ non-T cells from *S*. *japonicum*-infected mice was analyzed by FCM at indicated time points post-infection. Representative histograms illustrating PD-1 expression on CD8^+^ T cells or CD3^-^ non-T cells. (B) Bar graphs represent means ± SD of 15 mice from three independent experiments. ***P < 0.001.
(TIF)

S3 Fig CD4^+^ T cells tend to be anergic in *S*. *japonicum* infection. (A) Fas or PD-L1 expression on splenic or mesenteric CD4^+^ T cells from *S*. *japonicum*-infected mice eight weeks post-infection was analyzed by FCM. Representative histograms illustrating Fas or PD-L1 expression on CD4^+^ T cells. (B) Bar graphs represent means ± SD of 12 mice from three independent experiments. *P < 0.05, **P < 0.01, ***P < 0.001.
(TIF)

S4 Fig The total number of IL-4-producing CD4^+^ T cells is increased in the spleens or LNs of *S*. *japonicum*-infected mice with blockade of PD-1. The bar graphs show the absolute number of IL-4-, IFN-γ-, IL-17A-producing CD4^+^ T cells (A-C) or Treg cells (D) in splenic or mesenteric cells from *S*. *japonicum*-infected mice treated with anti-PD-1 mAb, control rat IgG2a or PBS. The absolute numbers of IL-4^+^/IFN-γ^+^/IL-17A^+^ CD4^+^ T cells or Treg cells were calculated as following: total cell number of the splenic or mesenteric cells × (frequency of CD4^+^ T cells in total cells) × (frequency of IL-4^+^/IFN-γ^+^/IL-17A^+^ cells or Treg cells in total CD4^+^ T cells). The data are expressed as the means ± SD of 15 mice from three independent experiments. **P < 0.01.
(TIF)

S5 Fig PD-1 blockade induces higher frequency of IL-4-producing hepatic CD4^+^ T cells in *S*. *japonicum*-infected mice. Representative staining (A) and mean percentages (B) for IL-4-, IFN-γ-, or IL-17A-producing CD4^+^ T cells or Treg cells from the livers of *S*. *japonicum*-infected mice treated with anti-PD-1 mAb, control rat IgG2a, or PBS. The data are expressed as the means ± SD of 15 mice from three independent experiments. **P < 0.01.
(TIF)

S6 Fig PD-1 blockade does not affect proportions of aTreg or rTreg cells in *S*. *japonicum*-infected mice. (A) Representative staining for CD62L^low^CD44^hi^ aTreg and CD62L^hi^CD44^low^ rTreg cells from the spleens or LNs of *S*. *japonicum*-infected mice treated with anti-PD-1 mAb, control rat IgG2a, or PBS. (B) The bar graphs show the average percentages of CD62L^low^CD44^hi^ aTreg and CD62L^hi^CD44^low^ rTreg cells within total Treg cells. The data are expressed as the means ± SD of 15 mice from three independent experiments.
(TIF)

S7 Fig PD-1 blockade does not affect Th2 differentiation after *S*. *japonicum* infection. (A) Representative staining for GATA-3 and PD-1 expression of CD4^+^ T cells from the spleens or LNs of *S*. *japonicum*-infected mice treated with anti-PD-1 mAb, control rat IgG2a, or PBS. (B) The bar graph shows the average percentages of GATA-3^+^ cells within total splenic or mesenteric CD4^+^ T cells. (C) The bar graph shows the average percentages of PD-1^+^ cells within splenic or mesenteric GATA-3^+^CD4^+^ T cells. The data are expressed as the means ± SD of 15 mice from three independent experiments.
(TIF)

S8 Fig Blockade of PD-1 increases the frequency of M2 macrophages in *S*. *japonicum*-infected mice. Liver Kupffer cells were purified from *S*. *japonicum*-infected mice treated with anti-PD-1 mAb, control rat IgG2a, or PBS. Expression of CD206 (M2 macrophages) on F4/80^+^CD11b^+^ macrophages was analyzed by FCM. Histograms are representative of three independent experiments and gated on F4/80^+^CD11b^+^ macrophages.
(TIF)

S1 Text Supporting text. This file contains detailed materials and methods for immunofluorescence staining and flow cytometry in supplementary figures.
(DOC)

S1 Checklist STROBE Checklist. (DOC)
