# Supplementary figures and images for: Blockade of PD-1 Signaling Enhances Th2 Cell Responses and Aggravates Liver Immunopathology in Mice with Schistosomiasis japonica
Source: PLoS Negl Trop Dis. 2016 Oct 28;10(10):e0005094. doi: 10.1371/journal.pntd.0005094 (PMC5085144; doi:10.1371/journal.pntd.0005094)

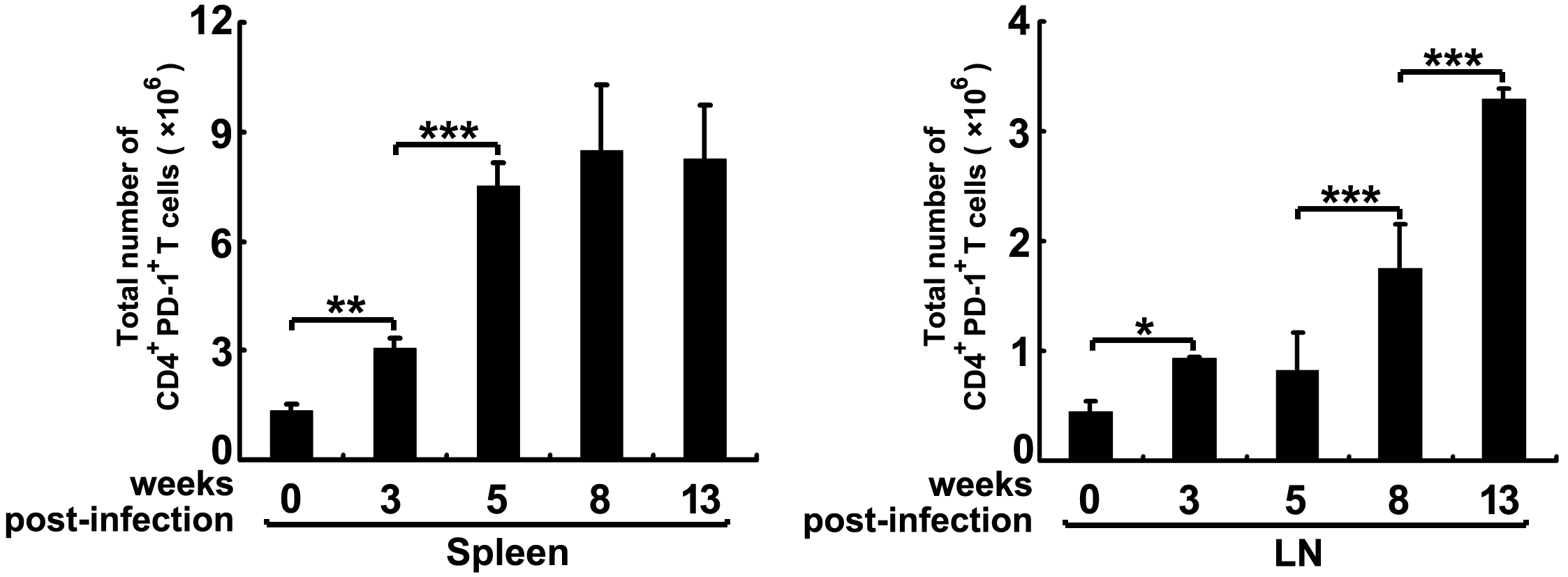

Supplement: S1 Fig — The bar graphs show the absolute number of PD-1+CD4+ T cells in splenic or mesenteric cells from mice at indicated time points after S. japonicum infection. The absolute numbers of PD-1+CD4+ T cells were calculated as following: total cell number of the splenic or mesenteric cells × (frequency of CD4+ T cells in total cells) × (frequency of PD-1+ cells in total CD4+ T cells). The data are expressed as the means ± SD of 15 mice from three independent experiments. *P < 0.05, **P < 0.01, ***P < 0.001. (TIF) [file pntd.0005094.s001.tif]

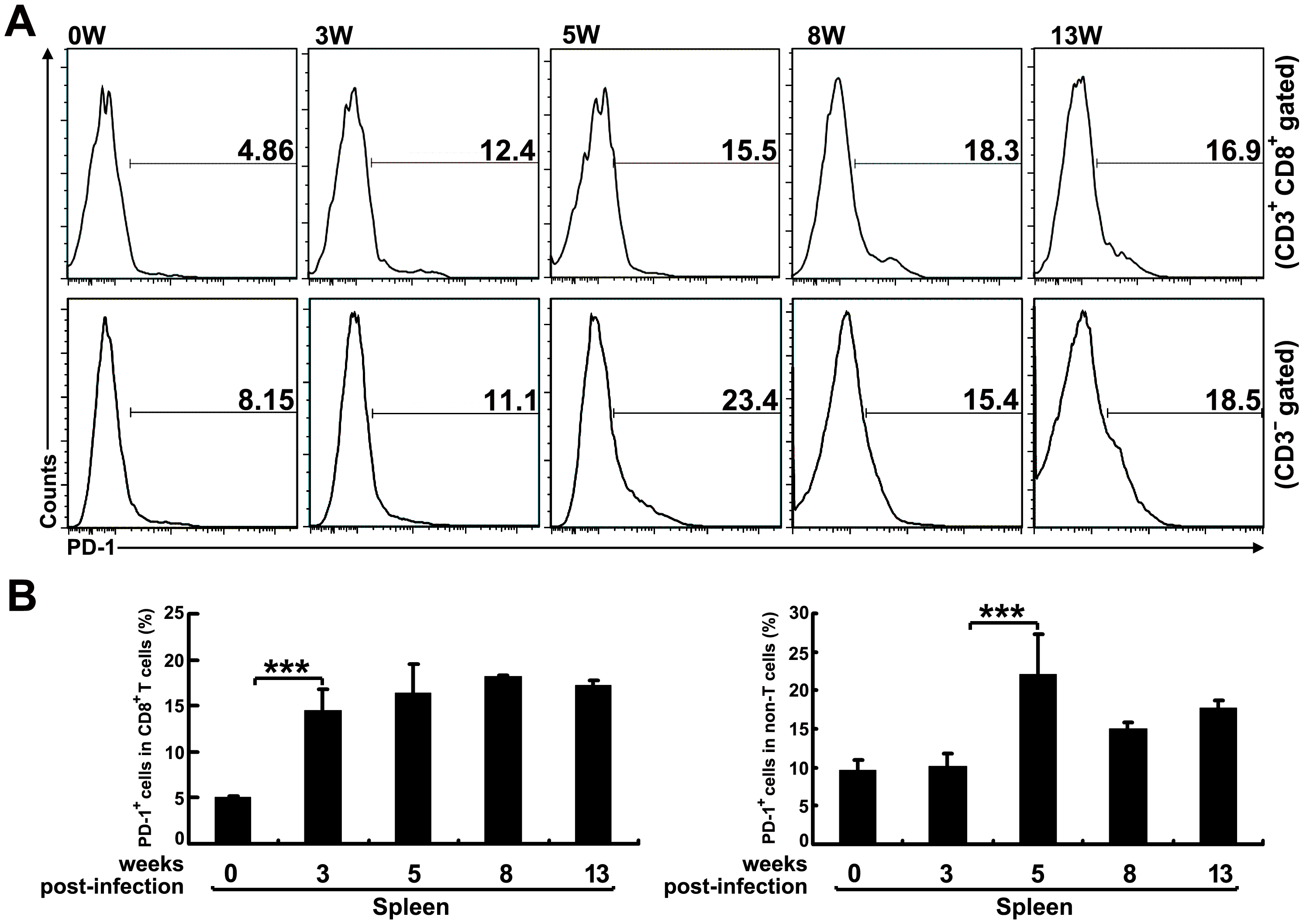

Supplement: S2 Fig — (A) PD-1 expression on CD8+ T cells or CD3- non-T cells from S. japonicum-infected mice was analyzed by FCM at indicated time points post-infection. Representative histograms illustrating PD-1 expression on CD8+ T cells or CD3- non-T cells. (B) Bar graphs represent means ± SD of 15 mice from three independent experiments. ***P < 0.001. (TIF) [file pntd.0005094.s002.tif]

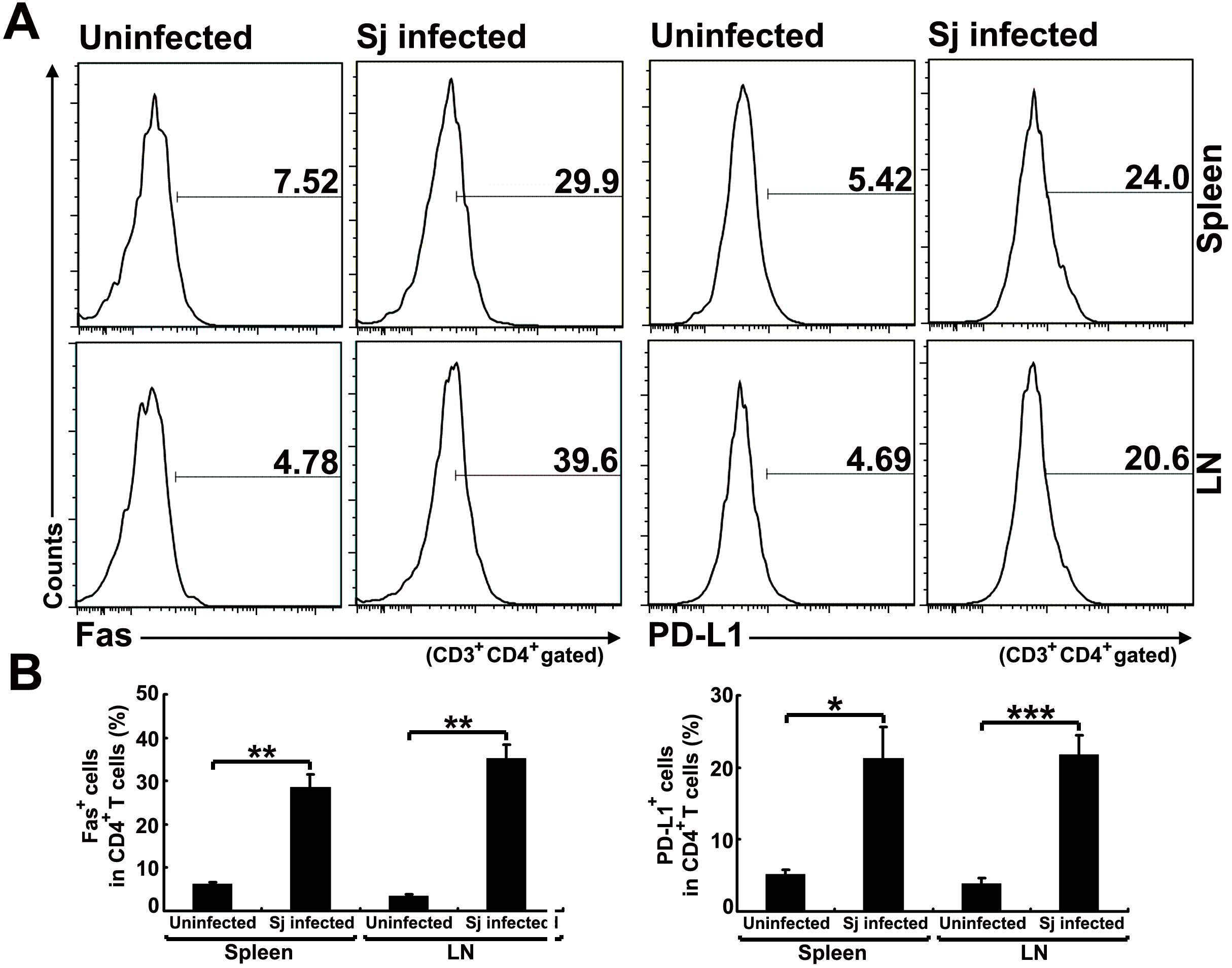

Supplement: S3 Fig — (A) Fas or PD-L1 expression on splenic or mesenteric CD4+ T cells from S. japonicum-infected mice eight weeks post-infection was analyzed by FCM. Representative histograms illustrating Fas or PD-L1 expression on CD4+ T cells. (B) Bar graphs represent means ± SD of 12 mice from three independent experiments. *P < 0.05, **P < 0.01, ***P < 0.001. (TIF) [file pntd.0005094.s003.tif]

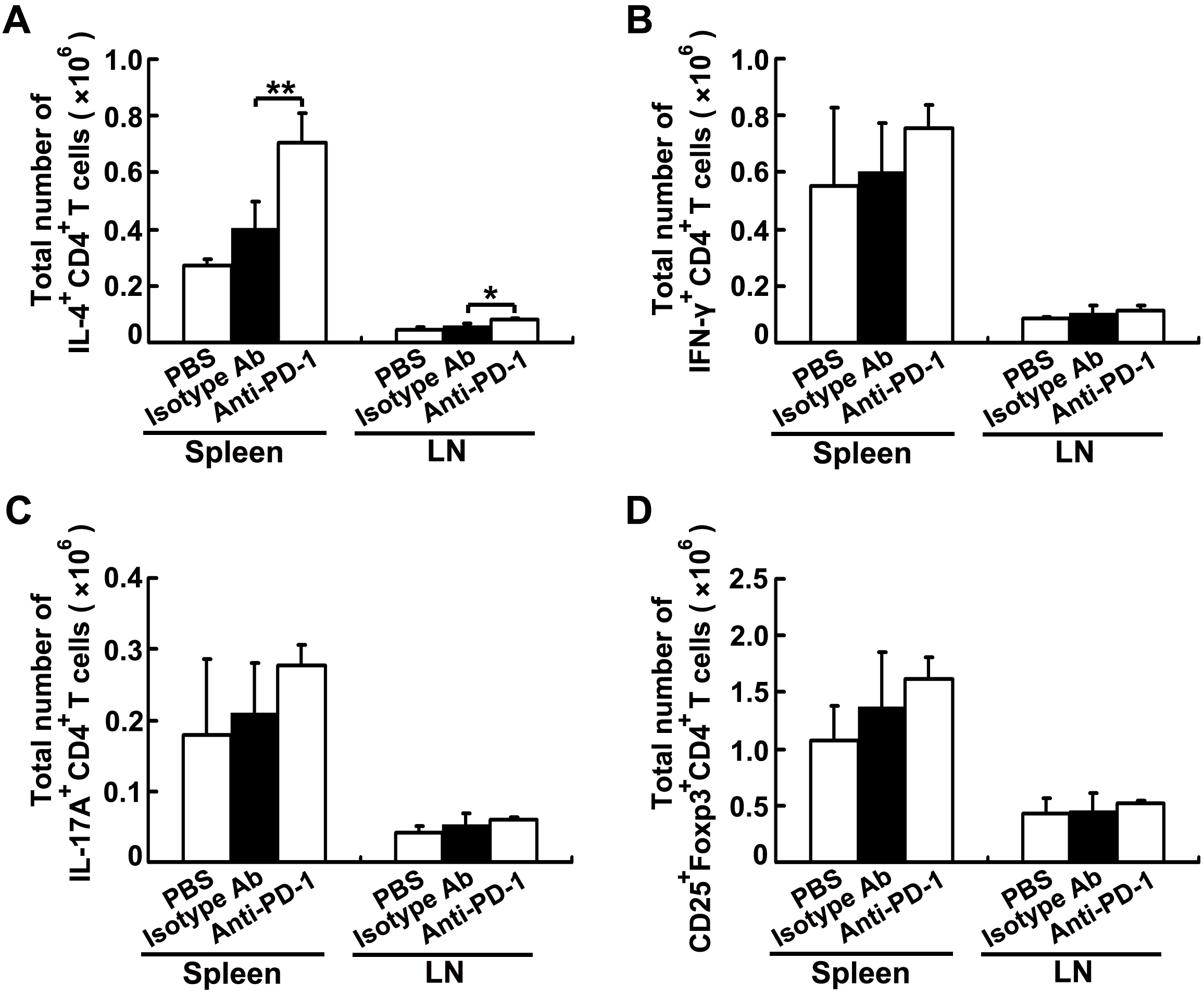

Supplement: S4 Fig — The bar graphs show the absolute number of IL-4-, IFN-γ-, IL-17A-producing CD4+ T cells (A-C) or Treg cells (D) in splenic or mesenteric cells from S. japonicum-infected mice treated with anti-PD-1 mAb, control rat IgG2a or PBS. The absolute numbers of IL-4+/IFN-γ+/IL-17A+ CD4+ T cells or Treg cells were calculated as following: total cell number of the splenic or mesenteric cells × (frequency of CD4+ T cells in total cells) × (frequency of IL-4+/IFN-γ+/IL-17A+ cells or Treg cells in total CD4+ T cells). The data are expressed as the means ± SD of 15 mice from three independent experiments. **P < 0.01. (TIF) [file pntd.0005094.s004.tif]

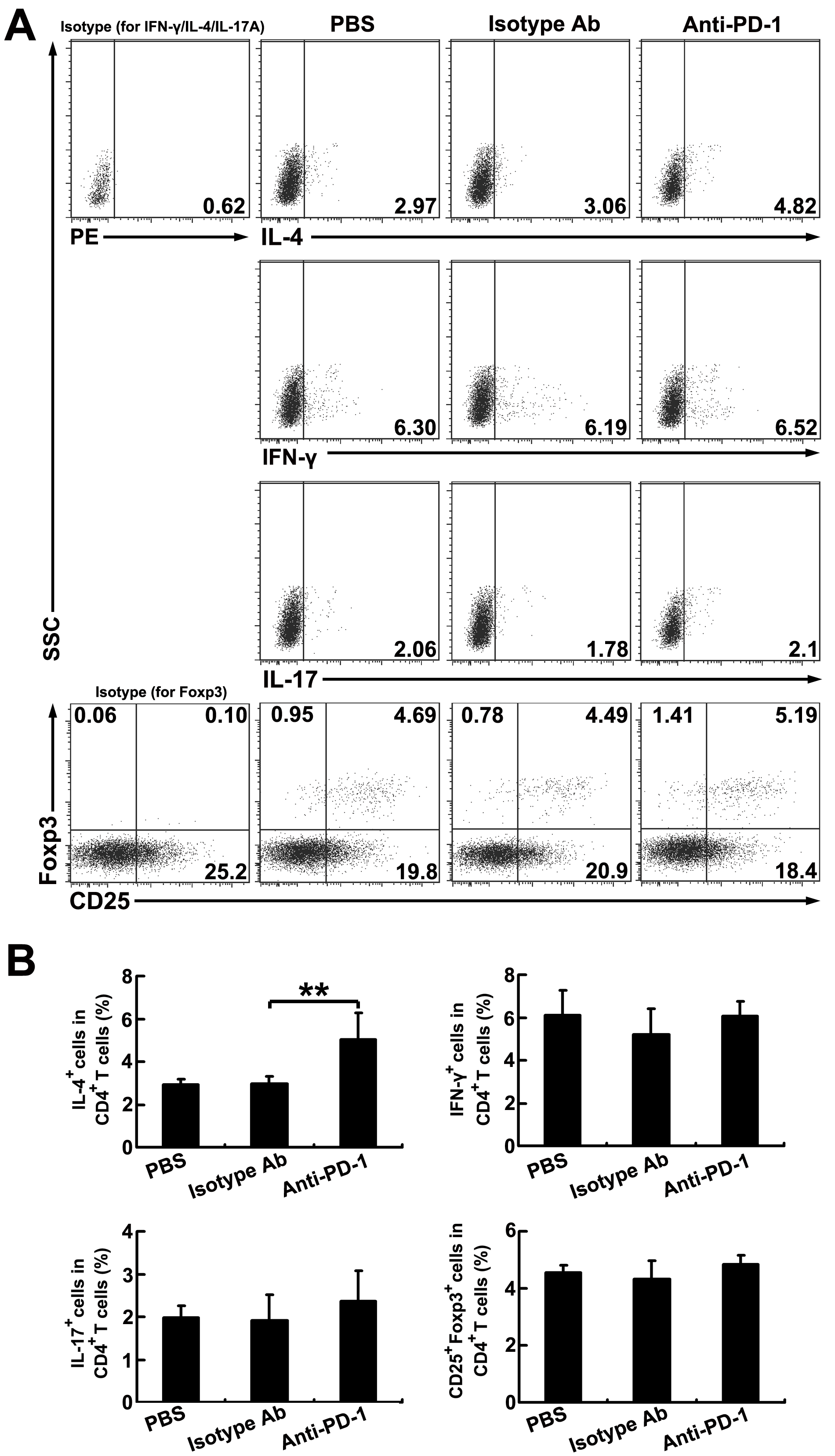

Supplement: S5 Fig — Representative staining (A) and mean percentages (B) for IL-4-, IFN-γ-, or IL-17A-producing CD4+ T cells or Treg cells from the livers of S. japonicum-infected mice treated with anti-PD-1 mAb, control rat IgG2a, or PBS. The data are expressed as the means ± SD of 15 mice from three independent experiments. **P < 0.01. (TIF) [file pntd.0005094.s005.tif]

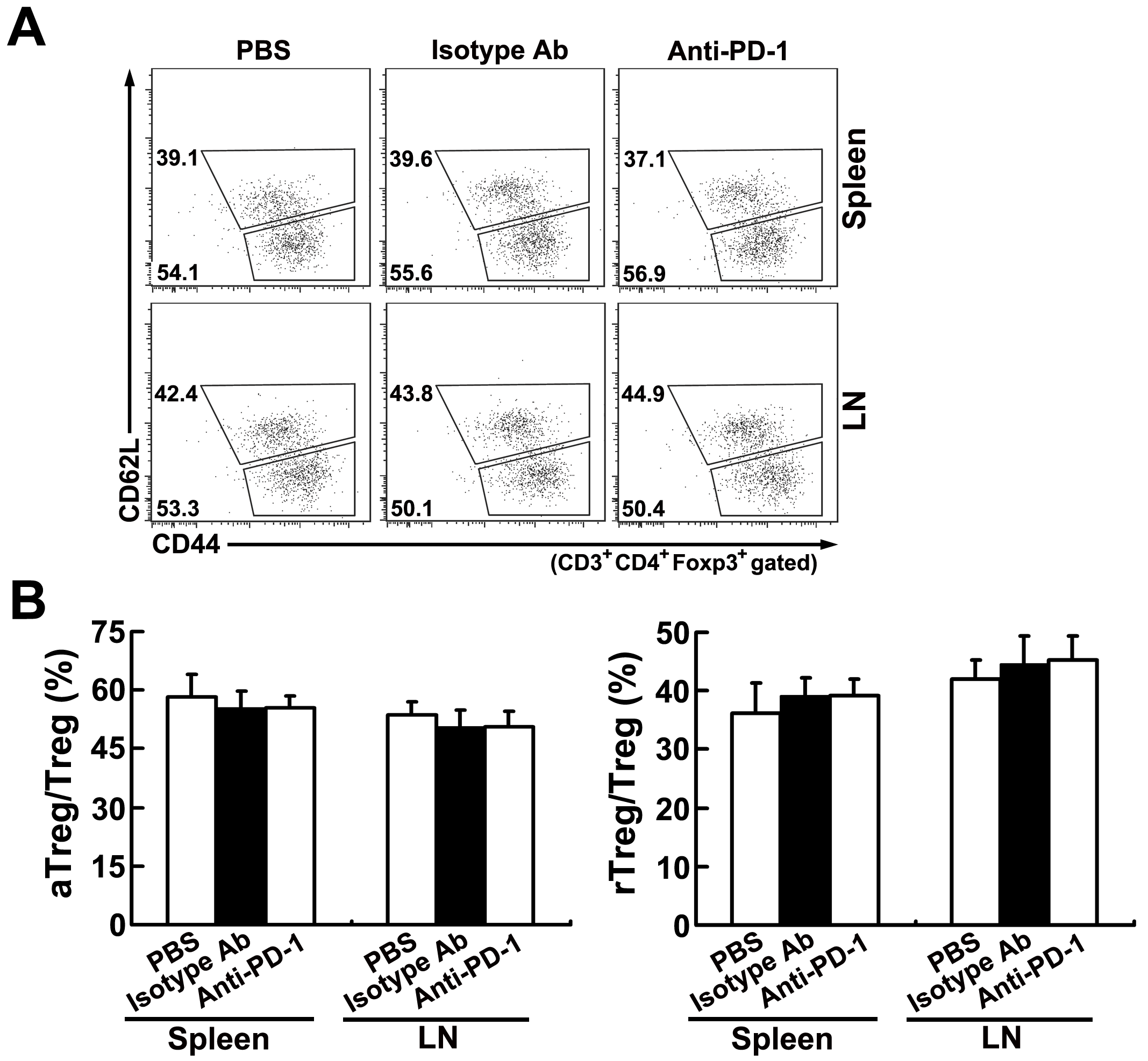

Supplement: S6 Fig — (A) Representative staining for CD62LlowCD44hi aTreg and CD62LhiCD44low rTreg cells from the spleens or LNs of S. japonicum-infected mice treated with anti-PD-1 mAb, control rat IgG2a, or PBS. (B) The bar graphs show the average percentages of CD62LlowCD44hi aTreg and CD62LhiCD44low rTreg cells within total Treg cells. The data are expressed as the means ± SD of 15 mice from three independent experiments. (TIF) [file pntd.0005094.s006.tif]

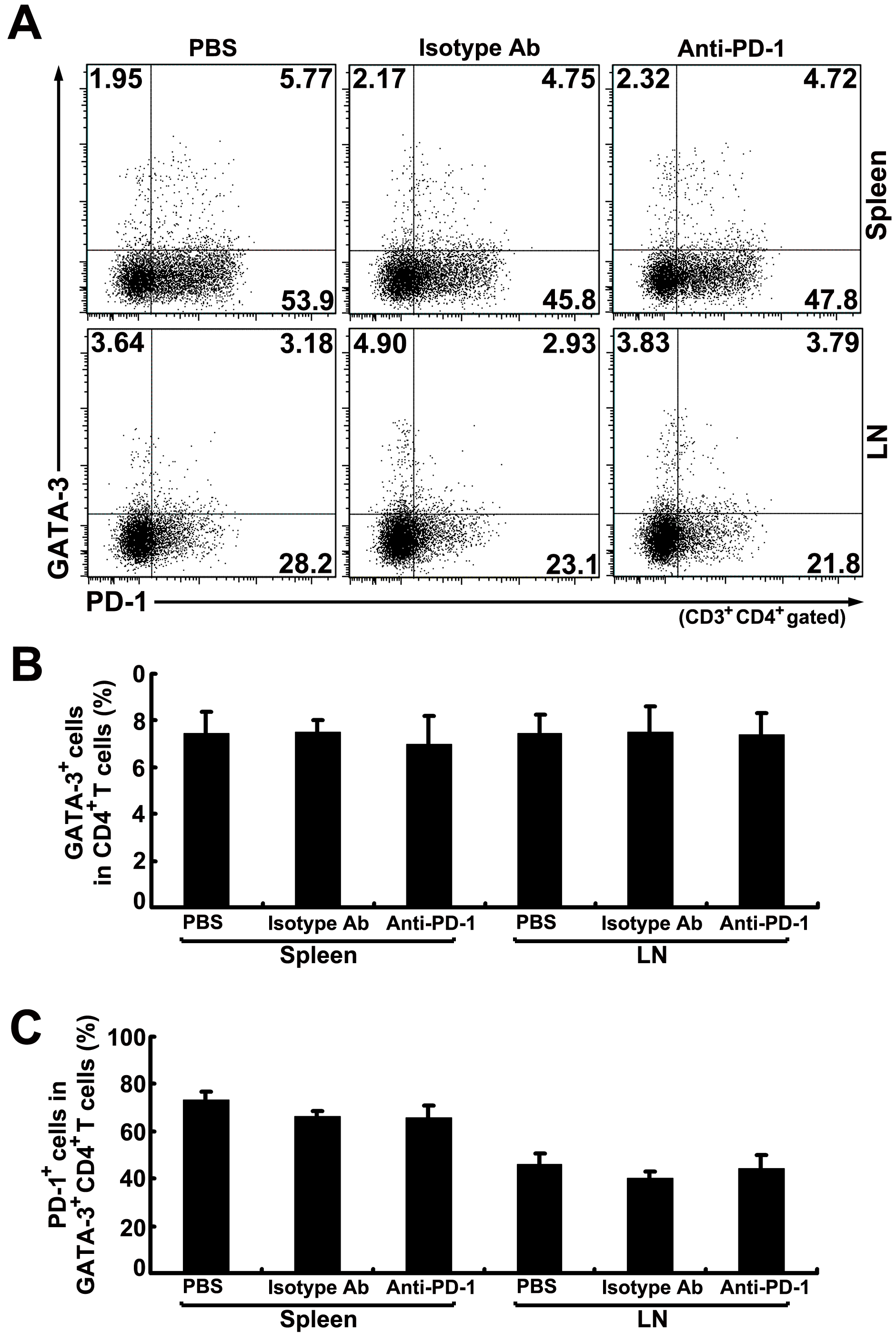

Supplement: S7 Fig — (A) Representative staining for GATA-3 and PD-1 expression of CD4+ T cells from the spleens or LNs of S. japonicum-infected mice treated with anti-PD-1 mAb, control rat IgG2a, or PBS. (B) The bar graph shows the average percentages of GATA-3+ cells within total splenic or mesenteric CD4+ T cells. (C) The bar graph shows the average percentages of PD-1+ cells within splenic or mesenteric GATA-3+CD4+ T cells. The data are expressed as the means ± SD of 15 mice from three independent experiments. (TIF) [file pntd.0005094.s007.tif]

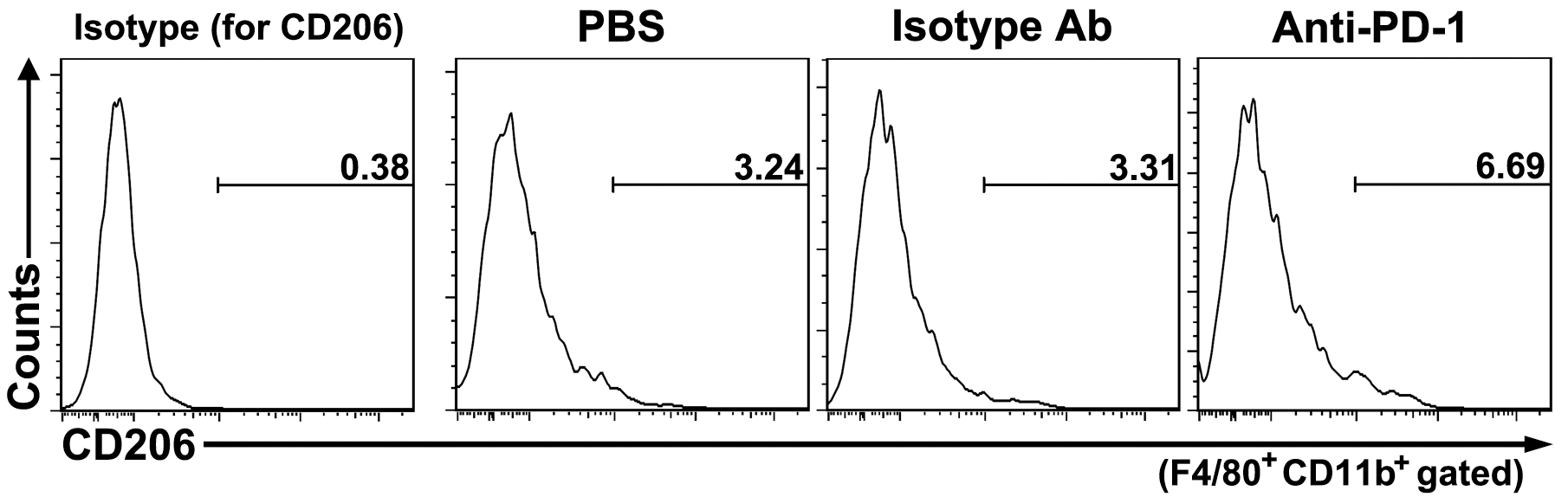

Supplement: S8 Fig — Liver Kupffer cells were purified from S. japonicum-infected mice treated with anti-PD-1 mAb, control rat IgG2a, or PBS. Expression of CD206 (M2 macrophages) on F4/80+CD11b+ macrophages was analyzed by FCM. Histograms are representative of three independent experiments and gated on F4/80+CD11b+ macrophages. (TIF) [file pntd.0005094.s008.tif]
