# Supplementary material for: Blockade of PD-1 Signaling Enhances Th2 Cell Responses and Aggravates Liver Immunopathology in Mice with Schistosomiasis japonica
Source: PLoS Negl Trop Dis. 2016 Oct 28;10(10):e0005094. doi: 10.1371/journal.pntd.0005094 (PMC5085144; doi:10.1371/journal.pntd.0005094)
Supplement: S1 Text — This file contains detailed materials and methods for immunofluorescence staining and flow cytometry in supplementary figures. (DOC) [file pntd.0005094.s009.doc]

**Supporting Information**

**Supplementary materials and methods**

**Immunofluorescence staining and flow cytometry (FCM)**

Liver Kupffer cells were separated from mice by Percoll gradient as previously described . Single-cell suspensions of splenocytes, lymph node cells or hepatic mononuclear cells were stained with the following preconjugated antibodies (all from eBioscience): CD3-APC/PerCP-Cy5.5 (clone 145-2C11), CD8-FITC (clone 53-6.7), PD-1-PE/PE-Cy7 (clone J43), Fas-PE (clone 15A7), PD-L1-PerCP-eFlour 710 (clone MIH5), CD62L-PerCP-Cy5.5 (clone MEL-14), CD44-PE-Cy7 (clone IM7), F4/80-FITC (clone BM8), CD11b-APC (clone M1/70), CD206-PE (clone C068C2), Foxp3-PE (clone FJK16s), and GATA-3-PE-Cy7 (clone TWAJ). Intranuclear staining for Foxp3 and GATA-3 was carried out using Foxp3 fixation/permeabilization buffers (eBioscience). Following immunofluorescence staining, the cells were examined using a FACSCalibur instrument (BD Bioscience) and analyzed using FlowJo (Tree Star, version 10.0.7).

**References**

**1. Hritz I, Mandrekar P, Velayudham A, Catalano D, Dolganiuc A, Kodys K, et al. (2008) The critical role of toll-like receptor (TLR) 4 in alcoholic liver disease is independent of the common TLR adapter MyD88. Hepatology 48: 1224-1231.**
