# Supplementary material for: Blockade of PD-1 Signaling Enhances Th2 Cell Responses and Aggravates Liver Immunopathology in Mice with Schistosomiasis japonica
Source: PLoS Negl Trop Dis. 2016 Oct 28;10(10):e0005094. doi: 10.1371/journal.pntd.0005094 (PMC5085144; doi:10.1371/journal.pntd.0005094)
Supplement: S1 Checklist — (DOC) [file pntd.0005094.s010.doc]

STROBE Statement—Checklist of items that should be included in reports of ***case-control studies***

|  | Item No | Recommendation |
| --- | --- | --- |
| **Title and abstract**  (Manuscript page 2 lines 2-13) | 1 | (*a*) Indicate the study’s design with a commonly used term in the title or the abstract |
|  |  | (*b*) Provide in the abstract an informative and balanced summary of what was done and what was found |
| Introduction | | |
| Background/rationale  (Manuscript page 4 lines 14-22 and page 5 line 1) | 2 | Explain the scientific background and rationale for the investigation being reported |
| Objectives  (Manuscript page 5 lines 1-9) | 3 | State specific objectives, including any prespecified hypotheses |
| Methods | | |
| Study design  (Manuscript page 6 lines 9-14 and 17-21) | 4 | Present key elements of study design early in the paper |
| Setting  (Manuscript page 6 lines 16-22 and page 7 lines 1-4) | 5 | Describe the setting, locations, and relevant dates, including periods of recruitment, exposure, follow-up, and data collection |
| Participants  (Manuscript page 6 lines 16-22 and page 7 lines 1-4) | 6 | (*a*) Give the eligibility criteria, and the sources and methods of case ascertainment and control selection. Give the rationale for the choice of cases and controls |
|  |  | (*b*) For matched studies, give matching criteria and the number of controls per case |
| Variables  (Manuscript page 6 lines 16-22 and page 7 lines 1-4) | 7 | Clearly define all outcomes, exposures, predictors, potential confounders, and effect modifiers. Give diagnostic criteria, if applicable |
| Data sources/ measurement  (Manuscript page 12 lines 19-23 and page 13 lines 1-3) | 8* | For each variable of interest, give sources of data and details of methods of assessment (measurement). Describe comparability of assessment methods if there is more than one group |
| Bias  (Manuscript page 6 lines 16-22 and page 7 lines 1-4) | 9 | Describe any efforts to address potential sources of bias |
| Study size  (Manuscript page 6 lines 17-21,) | 10 | Explain how the study size was arrived at |
| Quantitative variables  (Manuscript page 12 lines 19-23 and page 13 lines 1-3) | 11 | Explain how quantitative variables were handled in the analyses. If applicable, describe which groupings were chosen and why |
| Statistical methods  (Manuscript page 12 lines 19-23 and page 13 lines 1-3) | 12 | (*a*) Describe all statistical methods, including those used to control for confounding |
|  |  | (*b*) Describe any methods used to examine subgroups and interactions |
|  |  | (*c*) Explain how missing data were addressed |
|  |  | (*d*) If applicable, explain how matching of cases and controls was addressed |
|  |  | (*e*) Describe any sensitivity analyses |
| Results | | |
| Participants  (Manuscript page 14 lines 3-7, Table 1) | 13* | (a) Report numbers of individuals at each stage of study—eg numbers potentially eligible, examined for eligibility, confirmed eligible, included in the study, completing follow-up, and analysed |
|  |  | (b) Give reasons for non-participation at each stage |
|  |  | (c) Consider use of a flow diagram |
| Descriptive data  (Manuscript page 14 lines 5-7, Table 1) | 14* | (a) Give characteristics of study participants (eg demographic, clinical, social) and information on exposures and potential confounders |
|  |  | (b) Indicate number of participants with missing data for each variable of interest |
| Outcome data  (Manuscript page 14 lines 8-15) | 15* | Report numbers in each exposure category, or summary measures of exposure |
| Main results  (Manuscript page 14 lines 8-15) | 16 | (*a*) Give unadjusted estimates and, if applicable, confounder-adjusted estimates and their precision (eg, 95% confidence interval). Make clear which confounders were adjusted for and why they were included |
|  |  | (*b*) Report category boundaries when continuous variables were categorized |
|  |  | (*c*) If relevant, consider translating estimates of relative risk into absolute risk for a meaningful time period |

| Other analyses | 17 | Report other analyses done—eg analyses of subgroups and interactions, and sensitivity analyses |
| --- | --- | --- |
| Discussion | | |
| Key results  (Manuscript page 19 lines 14-16) | 18 | Summarise key results with reference to study objectives |
| Limitations | 19 | Discuss limitations of the study, taking into account sources of potential bias or imprecision. Discuss both direction and magnitude of any potential bias |
| Interpretation  (Manuscript page 19 lines 16-22) | 20 | Give a cautious overall interpretation of results considering objectives, limitations, multiplicity of analyses, results from similar studies, and other relevant evidence |
| Generalisability | 21 | Discuss the generalisability (external validity) of the study results |
| Other information | | |
| Funding  “This work was supported by grants from the Jiangsu Provincial Natural Science Foundation of China (BK20130896), the National Natural Science Foundation of China (No. 81501766) and the Natural Science Foundation of the Jiangsu Higher Education Institutions of China (15KJB310007) to Sha Zhou, and the grant from National Natural Science Foundation of China (No. 81271861) to Chuan Su.” | 22 | Give the source of funding and the role of the funders for the present study and, if applicable, for the original study on which the present article is based |

*Give information separately for cases and controls.

**Note:** An Explanation and Elaboration article discusses each checklist item and gives methodological background and published examples of transparent reporting. The STROBE checklist is best used in conjunction with this article (freely available on the Web sites of PLoS Medicine at http://www.plosmedicine.org/, Annals of Internal Medicine at http://www.annals.org/, and Epidemiology at http://www.epidem.com/). Information on the STROBE Initiative is available at http://www.strobe-statement.org.
